# Rethinking Hominin Air Sac Loss in Light of Phylogenetically Meaningful Evidence

**DOI:** 10.1002/evan.70019

**Published:** 2025-09-18

**Authors:** Axel G. Ekström, Fotios Alexandros Karakostis, William D. Snyder, Steven Moran

**Affiliations:** ^1^ Institute of Biology University of Neuchâtel Neuchâtel Switzerland; ^2^ Speech, Music & Hearing, KTH Royal Institute of Technology Stockholm Sweden; ^3^ Centre for Cultural Evolution, Department of Psychology Stockholm University Stockholm Sweden; ^4^ Paleoanthropology, Institute for Archaeological Sciences, Department of Geosciences Eberhard Karls University of Tübingen Tübingen Germany; ^5^ Senckenberg Centre for Human Evolution and Palaeoenvironment Senckenberg Research Institute Tübingen Germany; ^6^ Integrative Prehistory and Archaeological Science University of Basel Basel Switzerland; ^7^ Early Prehistory and Quaternary Ecology, Department of Geosciences Eberhard Karls University of Tübingen Tübingen Germany; ^8^ Linguistic Research Infrastructure (LiRI) University of Zurich Zurich Switzerland

## Abstract

The evolution of laryngeal air sacs in hominins has been a subject of considerable debate, with particular attention given to the inferred presence of air sacs in *Australopithecus afarensis* and inferred absence in Middle and Upper Pleistocene hominins. We challenge several assumptions prevalent in relevant discourse and assert that (1) while exhibiting morphological similarity, it cannot be ruled out that relationships between hyoid morphology and air sac morphology in extant African great apes may reflect convergence; (2) while the only known *A. afarensis* hyoid exhibits “ape‐like” bulla, this feature may have persisted following the loss of air sacs, and not be indicative of their presence per se; (3) because there are currently only five known hominin hyoid bones represented in the fossil record (with a single specimen predating the Middle Pleistocene) the evidential basis for interpreting air sac presence or absence is minimal; and (4) inferences toward a role of sexual selection and communicative behavior in explicating the loss of air sacs in the hominin lineage are undermined by the atypical sexual dimorphism patterns in early hominins. We advocate for a cautious approach to interpreting hominin behavior and evolution which prioritizes data over speculation, and underscore the need for rigorous evidence when constructing evolutionary narratives about early hominin vocal anatomy and its evolution.

## 1 Introduction

All extant nonhuman great apes possess “lateral ventricular” air sacs [1, 2, 3, 4] (Table 1). Thus, while modern humans do not, it has been argued that ancestral hominins likely did as well [6, 7, 8, 9]. In the African great apes *— Pan* and *Gorilla —* the air sacs extend via the thyrohyoid membrane [2, 3, 4, 6, 10, 11, 12, 13, 14], but modern humans possess only vestigial laryngeal saccules [15]. Exactly when air sacs were lost in hominin evolution is not known, though it has been argued that their presence (or absence) may be inferred from fossil specimens by reference to the hyoid bone [7, 16].

**Table 1:** Air sac, hyoid, and sexual dimorphism (of air sac morphology) in African great apes and modern humans. Descriptions from [5].

Species	Air sacs	Hyoid	Sex dimorphism
*Gorilla*	Bilaterally asymmetrical air sacs extending under the chest and pectoral regions.	Bulla	Larger in males
*Pan*	Paired, lateral laryngeal sacs extending under the skin of the neck, axillae, and chest.	Bulla	Size differences attributable to body size
*Homo sapiens*	None	No bulla	n/a

In adult humans, the hyoid rests anteriorly at the base of the mandible, superiorly to the thyroid cartilage of the larynx. It is integral to normal functions of the masticatory complex such as swallowing, eating, and speaking [17, 18, 19, 20]. In the extant nonhuman African (but not Asian) great apes [6, 21] and in the juvenile *Australopithecus afarensis* specimen described by Alemseged et al. [7], the hyoid possesses a characteristic curved bulla, which in the extant apes provides an extension from the air sac complex into the hyoid body [2, 6]. The hyoids of modern *Homo sapiens* and Neandertals (*H. neanderthalensis*) [22] are flat and horseshoe‐shaped [6, 23]. Accordingly, a bulla‐shaped hyoid has been interpreted as indicative of air sacs in extinct hominins [6, 7, 24, 25]. Broadly, the literature on air sacs and their evolutionary relevance (e.g. [24, 25]) is based on four assumptions. They are as follows:

**A1:** A direct relationship exists between air sacs and the specific bulla hyoid morphology of living African apes (e.g. [6]).

**A2:** There was conservation of the nature and functionality of the bulla hyoid morphology across (hominid) taxa (e.g. [6]).

**A3:** Available fossil evidence may inform research about the evolutionary loss of air sacs (e.g. [7, 24]).

**A4:** Hominin sexual dimorphism, air sacs, and social/sexual behavior are, or may be, correlated (e.g. [24, 25]).

Below, we discuss each assumption in order and highlight the poverty of the relevant evidence in the context of potential hominin air sac loss. We conclude there is little evidence to support the presence of laryngeal air sacs in early hominins, and no definitive relationship exists between air sac evolution and hominin social organization, sexual dimorphism, or vocal anatomy.

## 2 Air Sacs, Extant Apes, and Extinct Apes: A Phylogenetically Narrow Problem

Only two extant genera of nonhuman African apes exist, *Pan* and *Gorilla*, with a notably sparse fossil record. However, at present there are no known hyoid specimens representing either any basal African ape, nor any ancestral member of the *Pan* or *Gorilla* lineages. Given the paucity of contradictory evidence, the bulla hyoid morphology remains the most parsimonious hypothesis for the ancestral condition of African apes. However, the example of knuckle‐walking serves as a cautionary illustration of how seemingly *synapomorphic* traits – those that appear to have evolved in a common ancestor of African apes and differentiate them from other apes – can instead arise through convergent evolution [26].

Specifically, it has been suggested that knuckle‐walking evolved independently in chimpanzees and gorillas due to similar ecological pressures despite their last common ancestor being potentially primarily arboreal [27]. Although it should be noted that this hypothesis is subject to extensive debate, this case highlights the ambiguity that can be present when drawing inferences about soft tissue anatomy and function, as well as behavior, from fossils (see discussion on undetermination between competing hypotheses and making inferences via comparisons between extant and extinct systems in [28, 29]). Similarly, the role of air sacs in vocal production and behavior remains the subject of uncertainty: it is possible that divergent functional roles evolved in the different African great ape lineages (e.g. [30]). Similarly, even assuming that some human ancestors did possess air sacs, their hypothetical presence across different genera does not necessarily imply identical form, function, or evolutionary origin.

## 3 Functional Interpretations: Hyoid Morphology and Air Sac Morphology

### 3.1 Potential Vestigiality and Exaptation

**Figure 1:**
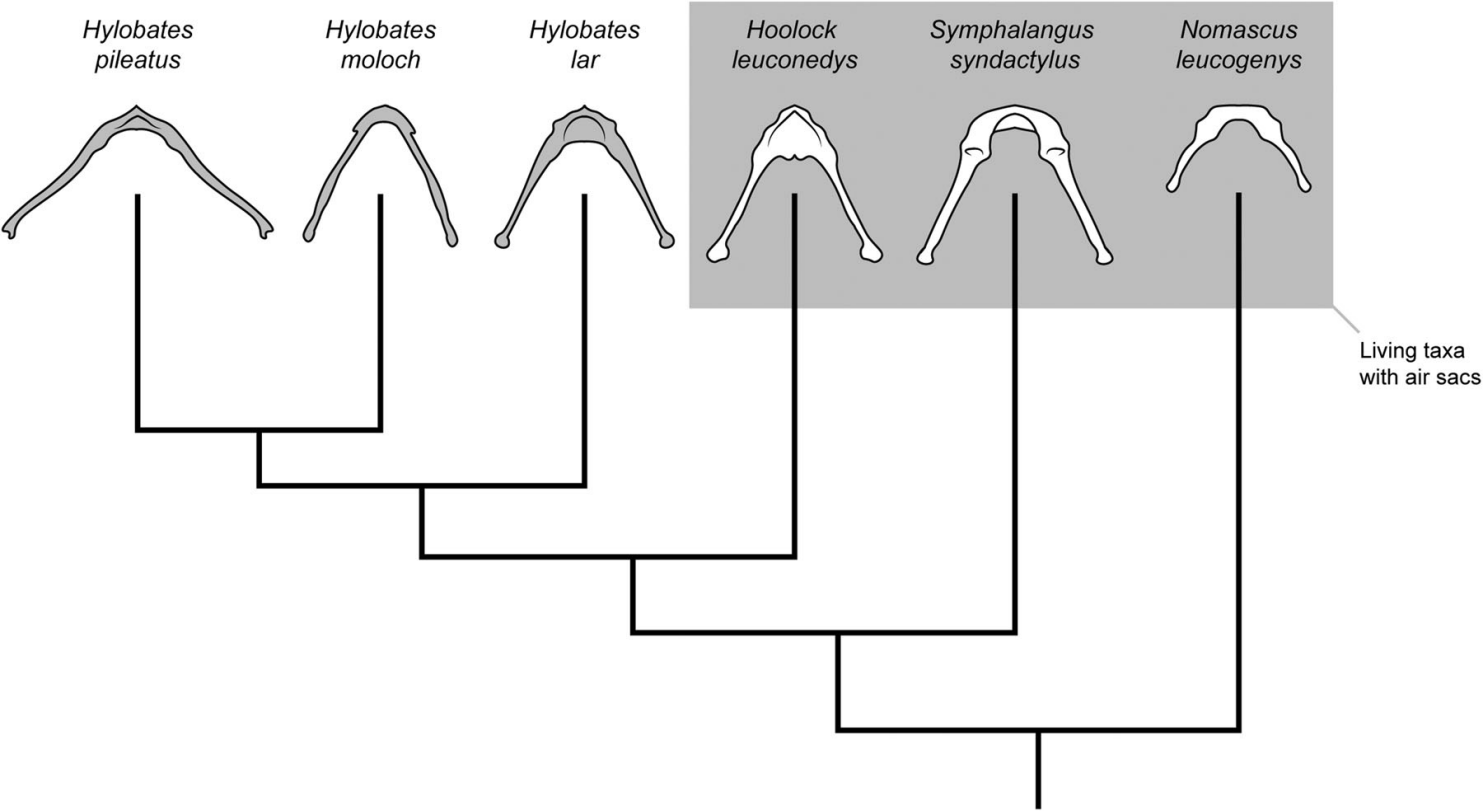
Hyoid morphology and hyoid‐air sac relationships across hylobatids. Adapted from Zihlman and Underwood [31].

Skeletal structures can persist long after the associated soft tissue anatomy has been lost, as demonstrated by well‐known examples such as vestigial hindlimbs in cetaceans. Accepting the hypothesis that humans evolved from an ancestor with an air sac, it remains possible that the loss of the bulla hyoid morphology occurred after the air sac itself was lost. This scenario bears consideration in the absence of specified strong selective pressures acting on the bulla hyoid structure. Consequently, the identification of a bulla hyoid morphology in a fossil hominin does not necessarily indicate the presence of an air sac at that evolutionary stage. In theory, a bulla hyoid morphology may have remained in hominins *after* air sacs were lost. Indeed, in considering more phylogenetically‐distant cases, we find indications of a complex history of air sacs having been gained, lost, and potentially even re‐gained numerous times among the primates [8], whereas hyoid morphology varies inconsistently across species with and without air sacs and among related species *post*‐air sac loss (see apparent variation among the hylobatids in Zihlman and Underwood [31]; Figure 1). Though this requires further systematic investigation, such ambiguities underscore the need for caution when interpreting fossil evidence, particularly when soft tissue features play a critical role in understanding evolutionary transitions.

The bulla hyoid morphology, likely associated with air sac attachment in ancestral apes, may have served a distinct function in certain transitional hominins. While its original role might have been related to vocalization or respiratory efficiency (e.g. [3, 6], cf [5]), this morphology may have undergone exaptation, having evolved for one purpose before acquiring a new different or complementary function in evolving lineages. Exaptation may have leaned into functions largely unrelated to vocal production, from adapting structural support within the changing hyo‐laryngeal complex [32, 33], or stabilizing deglutition and head movement [17, 18, 34] following changes to the general shape of the airways [35].

**Figure 2:**
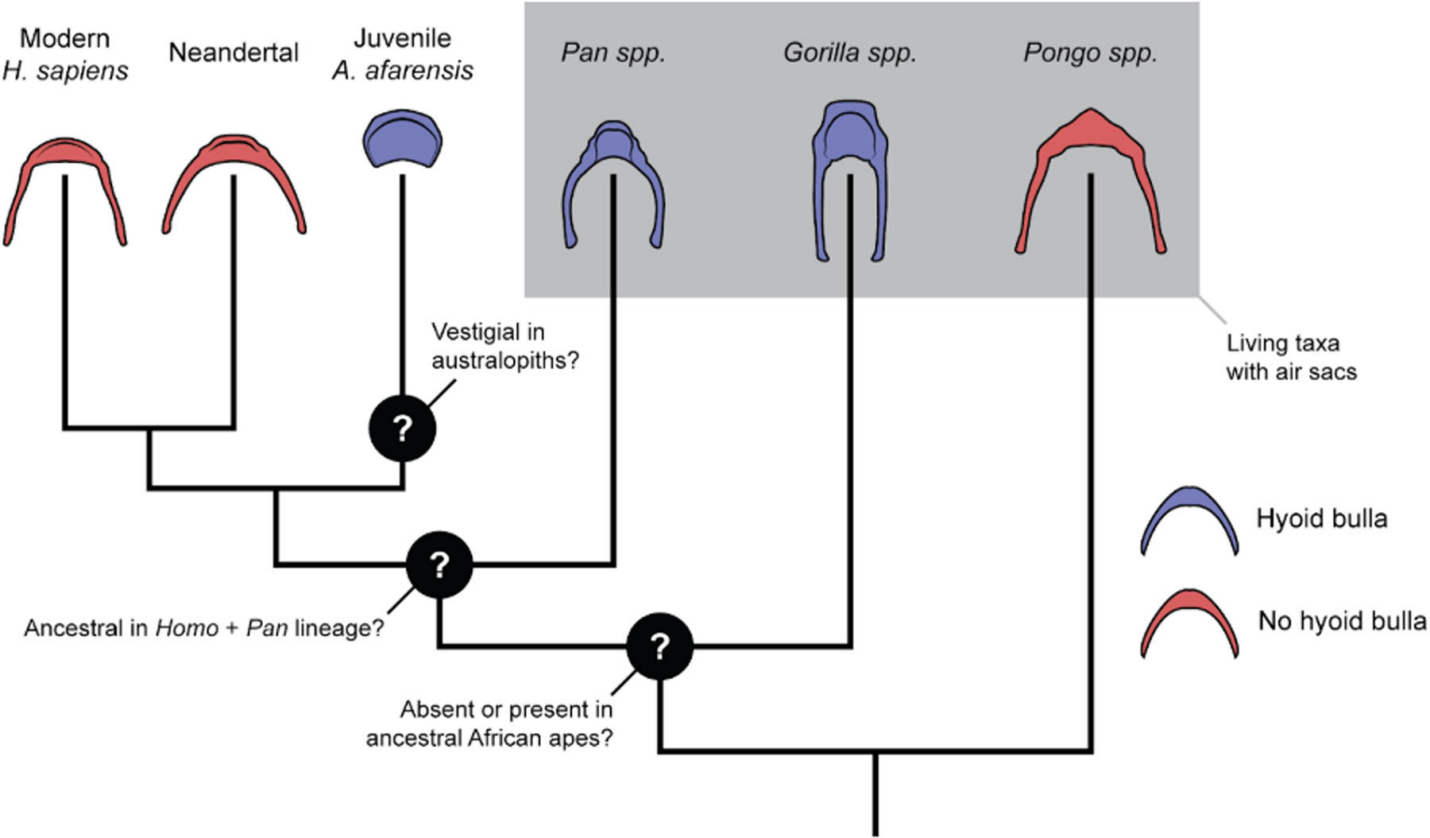
Ambiguous bulla hyoid‐air sac relationships across hominid taxa. Hyoid illustrations are idealized and symmetrical representations and not to scale.

Following this interpretation, retention of the bulla hyoid morphology may not indicate a persistence of laryngeal air sacs, but exaptation of hyoid function following the loss of its prior soft‐tissue relationship in early hominins. Alternatively, the bulla hyoid may have also been a vestigial feature in basal hominins, retained without a clear adaptive purpose. The ambiguity surrounding its function highlights the broader challenges in interpreting morphological traits in the fossil record, where the potential for both vestigiality and functional repurposing must be carefully considered (Figure 2). In this context, integrative biomechanical and morphological analyses should be conducted to investigate potential vestigiality of the hyoid and associated organs [36].

Future research may stand to greatly benefit from the application of 3D quantitative comparative methods across species, potentially relying on existing virtual datasets, such as microCT or MRI scans (e.g., see [37]). For instance, landmark‐based three‐dimensional (3D) geometric morphometrics (GM) offers a robust toolkit for analyzing hyoid morphological variation across species with differing functional demands (e.g. [38]). These methods may help in clarifying form–function relationships by identifying patterns of shape variation both independently of, and in relation to, species size (i.e., allometry), and disentangling developmental or biomechanical influences from scaling effects (e.g. [39]). Complementary methods, including the Validated Entheses‐based Reconstruction of Activity (VERA) approaches (e.g. [40, 41]), can be used to reconstruct habitual muscle and ligament use from skeletal remains, helping to link soft tissue function with hyoid bone morphology. Virtual 3D data can also be integrated into advanced biomechanical musculoskeletal models that simulate hyoid movement under varying muscle and ligament parameters [36, 42]. Such simulations may provide a more direct basis for evaluating the relationship between hyoid form and potential vestigiality within the context of surrounding soft tissue structures and their combined function.

Finally, in the vocal domain, effects of air sacs have been asserted [24] but never investigated outright in living great apes (cf [30, 43]). For example [24] claims that the presence of air sacs in a primate vocal tract introduces “a new resonance near the resonance frequency of the isolated air sac appears and acoustic energy at this frequency is radiated effectively through the air sac wall, thus increasing acoustic output at this frequency” [24]. Resonance frequencies are here treated as functionally equivalent with *formants*, defined as peaks in acoustic spectra, generally held to correspond to vocal tract shape ([44, 45]). By manipulating the shape of their vocal tract, speakers navigate their “vowel space”—operationalized as a two‐dimensional representation of first‐second and first‐third formant pairs (e.g., [44, 46]). Functionally, the presence of an additional low‐frequency formant (hypothesized for nonhuman great apes and early hominins) would constrain one dimension of this “space”, impairing vowel production capacities (i.e., the range of available vowel sounds).

However, de Boer's determination of the relevance of air sac loss to hominin vocal communication was based on *Symphalangus syndactylus* and alouattines – both taxa characterized by highly derived air sac morphologies and unique hyoid‐air sac relationships [5, 47]. Whether a comparable constraint applies to African apes remains hypothetical and needs direct tests. Moving forward, extensive comparisons of respiratory ([8, 43]) and call production behaviors [30, 48, 49] across great ape species are necessary. The tentative articulatory‐acoustic scheme outlined by Grawunder et al. [49] suggests that, as in humans, a high jaw and pursed lips produces two low‐frequency formants close together (overlapping with [u], the vowel in “pool”), while a low jaw and open mouth produces two higher more widely spaced formants (overlapping with [a], the vowel in “mah”). This data suggests vowels in humans and vowel‐like calls in chimpanzees are effectively homologous, and that de Boer's [24] analysis overestimates the relevance of air sacs to hominin evolution. Complementary analyses will be necessary to verify this.

### 3.2 Ontogenetic Shape Changes to Hyoid Morphology

Studies of hyoid bone morphology in developing humans [50] and chimpanzees [13], (cited in [51]) show that hyoid bones may change in shape with development. Compared to modern humans, in other adult extant great apes, the hyoid is positioned higher relative to the vertebral column [11, 12, 13, 51, 52], but, as in humans, descends in relation to the vertebral column with maturity [12, 13, 53, 54]. From infancy to adulthood, the hyoid bone undergoes gradual ossification, starting as a small, cartilaginous structure with a relatively short and broad U‐shape, with separate components that slowly fuse and harden over time. The greater and lesser horns (cornua) are not fully developed or fused with the body of the bone. Throughout juvenile development, the U‐shape of the hyoid elongates as the greater horns lengthen relative to the body of the bone. The body becomes more prominent, and the lesser horns become more distinct and the overall curvature of the bone increases slightly. In adolescence, ossification continues, and the hyoid descends in the neck. By adulthood, the hyoid is fully ossified and maintains its mature shape through the lifespan.

Against such observed changes, inferring australopith adult hyoid morphology is problematic. Namely, the only australopith specimen that exists is that of a presumed female 3‐year‐old juvenile Dikika *A. afarensis* [7]. As such, at this time, we cannot rule out the potential changes to hyoid morphology in australopith maturation. The adult australopith hyoid, for which we currently possess no referent, may have been more comparable to that of extant African great apes, that of modern humans, or defined by its own set of characteristics. While we are not currently aware of any study of ontogenetic hyoidal changes in any species of *Gorilla* (cf [55, 56]. but see [2, 6, 33, 37, 51, 54]), such complementary work may shine light on possible divergence (or lack thereof) of hyoid attachments in *Gorilla* and *Pan*.

## 4 Scarcity of Fossil Hyoids

The hyoid is one of the least represented elements in the fossil record: the only known hominin specimens represent *A. afarensis* (*n* = 1) [7], *H. heidelbergensis* (*n* = 2) [9], and Neandertals (*H. neanderthalensis*) (*n* = 2) [22, 57]. Capasso et al. [58] reported an ostensible Homo erectus hyoid bone, which was later determined to be “better identified as the posterior rim of the first cervical vertebra” [59, p. 235]. This rarity of hyoid preservation in the fossil record is partly attributable to taphonomic biases. As a small and fragile bone embedded deep in the neck and surrounded by soft tissues, the hyoid is highly susceptible to post‐mortem disarticulation and degradation. This inherent preservation bias not only limits sample size but skews temporal and taxonomic representations of hyoids, impeding confident reconstruction of evolutionary trajectories. Addressing this challenge requires methodological strategies that go beyond reliance on fossil specimens alone. To this end, nonfossil‐based approaches such as vocal tract modeling (e.g. [24, 60, 61]) can offer valuable complementary tools. Such models can simulate the acoustic properties of vocal anatomy based on known or inferred anatomical parameters, providing testable predictions about vocal capabilities. Similarly, probabilistic frameworks like Bayesian phylogenetic inference may help assess trait evolution (e.g., air sac presence or vocal tract configuration) by modeling their distribution across living and extinct taxa under specified evolutionary scenarios (e.g. [62]). When integrated with fossil and comparative anatomical data, these approaches can provide a more robust, interdisciplinary foundation for understanding the functional evolution of the hyoid and the vocal tract more broadly.

The Dikika *A. afarensis* hyoid bone and the Sima de los Huesos hominins are separated by nearly 3 million years in geologic time [7, 63]. This period denotes one of the most remarkable stretches of time in hominin evolutionary history. Throughout this time window, hominins, having already transitioned to obligate terrestrial bipedality [27, 64, 65], experienced a dramatic increase in brain size [66, 67] and marked reduction of the face and jaw [54, 68], developed fully opposable and highly efficient thumbs [42, 69], transitioned from the rudimentary stone tools of the Oldowan cultural industry to the comparatively more demanding Acheulean industry of stone tool manufacture [70, 71, 72], developed cooking and complex processing of foods [73, 74] and dramatically expanded its geographical range [75, 76, 77]. The period also involved a great range of other possibly interacting factors [41, 42, 78, 79, 80, 81, 82, 83, 84, 85, 86, 87], which are not recognized in theories of air sac evolution.

**Figure 3:**
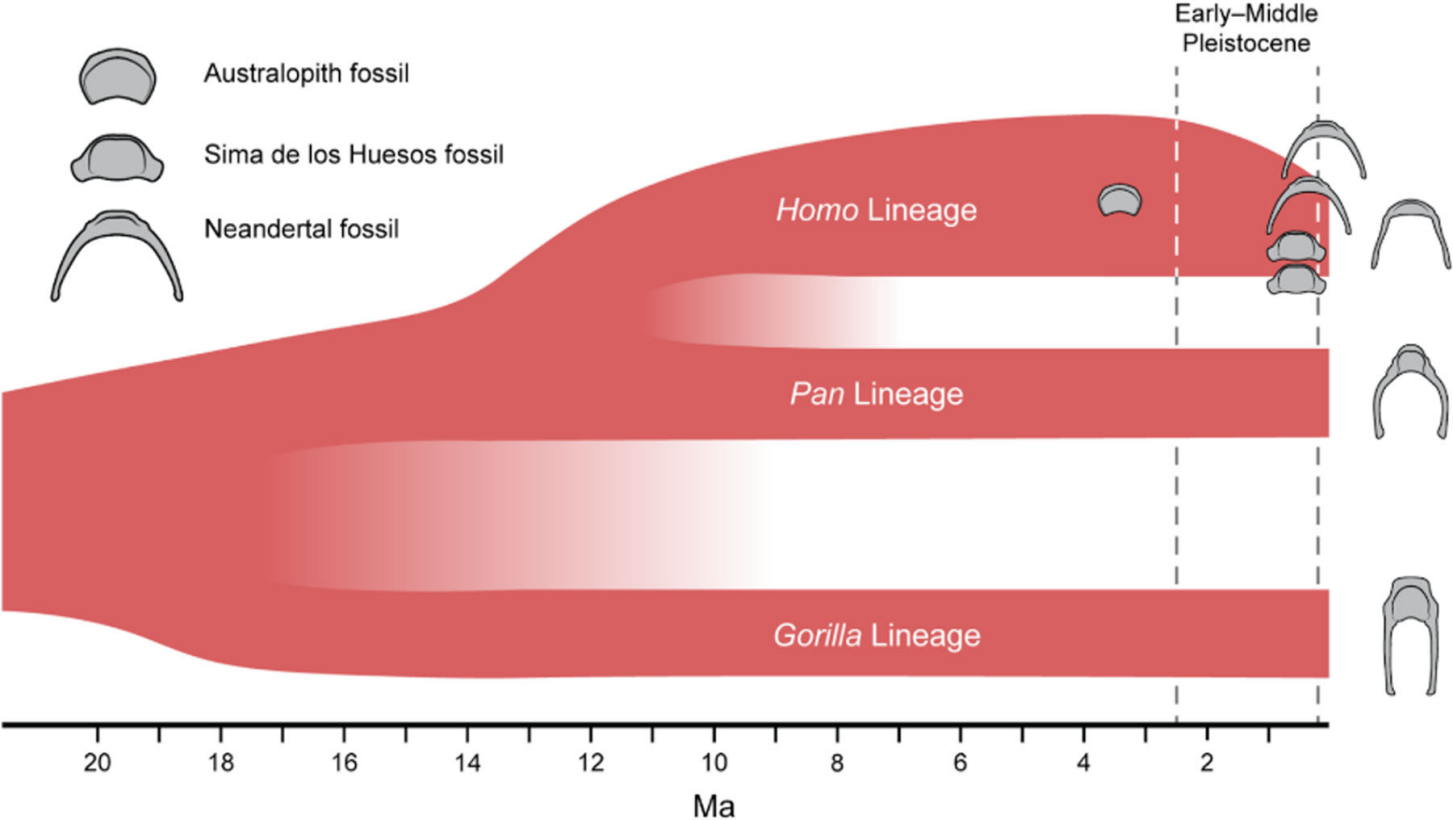
Timeline of known fossil hominin and African great ape hyoids. Hyoid illustrations are idealized, symmetrical representations and not to scale. Based on Langergraber et al. [88]. Approximate dates for each are ~3.3 Ma for the *A. afarensis* fossil (DIK 1/1); ~0.53 Ma for the Sima de los Huesos *H. heidelbergensis* fossils (AT‐1500, AT‐2000); and ~0.43 Ma and ~0.061 Ma for the Neandertal fossils (SDR‐034 and Kebara 2, respectively).

Indeed, the influence of such diverse and variable factors is potentially significant but cannot be realistically assessed due to the absence of hyoids across most of the fossil record (Figure 3, Table 2). While these transformative developments in hominin anatomy, technology, and behavior provide a context for appreciating the extent of possible interacting selection pressures, identifying specific anatomical markers related to speech and vocalization remains challenging. One possible such marker, the bony labyrinth—a complex, spiral‐shaped structure within the inner ear that houses the cochlea, vestibule, and semicircular canals, playing a critical role in hearing and balance—offers certain advantages over the hyoid bone as a line of evidence (cf [90]), but it also presents unique limitations.

**Table 2:** All known fossil hominin specimen hyoid bones as of 2025.

Name	Specimen	Hyoid bulla present	Species	Dating
DIK‐1/1^a^	Presumed juvenile female	✔	*A. afarensis*	~3.3 Ma
AT‐1500^b^	Hyoid body: Nearly complete	X	*H. heidelbergensis* e	~0.53 Ma
AT‐2000^b^	Hyoid body: Less complete than AT‐1500	X	*H. heidelbergensis* e	~0.53 Ma
SDR‐034^c^	Hyoid body	X	*H. neanderthalensis*	~0.43 Ma
Kebara 2^d^	Hyoid body (Male)	X	*H. neanderthalensis*	~0.061 Ma

^a^ Alemseged et al. [7]

^b^ Martínez et al. [9]

^c^ Cabo et al. [57]

^d^ Arensburg et al. [22]

^e^ The Sima de los Huesos hominins are now generally considered early Neandertals [89].

In particular, the bony labyrinth provides more specimens for analysis; in the comparative analysis conducted by Spoor et al. [91] alone, the sample comprised 24 labyrinths—all from the Middle Pleistocene or more recent epochs—of which a majority (*n* = 15) were Neandertal in origin. Additional such data is also available [92]. However, like hyoids, their utility is constrained by poor temporal resolution. Most existing specimens and research are heavily biased towards the late Middle to Late Pleistocene, which limits our understanding of earlier periods [93]. This is particularly problematic if the critical period for relevant selective pressures occurred during earlier periods.

Moreover, while the bony labyrinth offers insights into aspects of sensory and neural development, it does not provide direct information about speech production—an area where the hyoid bone holds more promise [54, 94, 95]. Each line of evidence carries specific drawbacks and potential insights, and a comprehensive approach requires a critical evaluation of these factors. Rather than relying on isolated hypotheses, such as hypotheses of air sac loss specifically, a more effective strategy would involve synthesizing multiple lines of evidence, ideally integrating both biological and cultural indications of behavioral advances (e.g. [87, 96, 97]). Ultimately, such an integrative approach is necessary to build a coherent and well‐supported narrative of hominin evolution—particularly those relating to speech and communicative behaviors more generally.

As summarized by [35], “the hyoid, basicranium, mandible, and cervical vertebrae all provide different insights into the evolution of the vocal tract and the timing of the appearance of hominin speech abilities.” Current hypotheses of air sac function have put air sacs at the forefront of theories (e.g., “loss of air sacs improved hominin speech abilities” [24]). We urge that, recognizing the substantial data limitations precluding far‐reaching conclusions, future work should consider the possible evolutionary roles of the organs in a comparative context [87]. Other anatomical features—including the vertebral column and spinal cord (e.g. [98, 99]) and the shape and dimensions of the airways and vocal tract [35, 94]—may yet yield important complementary insights.

## 5 Uniquely Hominin Sexual Dimorphism Compounds Problems of Interpretation

Dixson's [5] synthesis of primate anatomical data indicates a relationship between air sacs and mating systems: “Selection pressures have apparently operated on both sexes in many monogamous primates to favour the development of laryngeal specializations which enhance their loud calls […] Sexual dimorphism of laryngeal structure, coupled with a propensity for males alone to give loud calls or to give more pronounced calls than females, has evolved in various forest‐living primates which have polygynous or multimale‐multifemale mating systems.” Taking a similar stance [25], speculates, based on observations of howler monkeys (*Alouatta* spp) [100] that “variation in the size and shape of air sacs can be linked to variation in reproductive competition among species”.

The authors’ findings suggest that larger hyoids significantly lower resonant frequencies of the caller vocal tract. Because longer vocal tracts (and larger animals) will produce lower‐frequency resonants on average, this effect would convey an impression of greater size to the listener (e.g. [101, 102, 103, 104]). Resting on the assumption that air sacs play a part in intrasexual competition, Dunn [25] argues for a sexual selection hypothesis of the hominin loss of air sacs. Effectively, instead of relying on loud, deep calls to compete with other males, hominins might have developed alternative strategies, possibly including more complex vocal communication and social behaviors. This shift could have reduced the advantage of having air sacs, eventually leading to their loss.

However, available data is decidedly ambiguous for inferring any significant relationship in hominin evolution. Howler monkeys possess highly exaggerated air sacs and hyoids relative to body size, suggesting a disproportionate selection for these vocal anatomical features across species. As such, comparisons with such distantly related species appear questionable. The issue becomes even more complex when considering fossil hominin data.

Across primates, greater dimorphism generally indicates lower levels of male‐male competition across primates [5, 105]. In gorillas (*Gorilla spp*.), which have low levels of male‐male competition, males may be more than twice the size of females, while chimpanzees (*Pan troglodytes*) exhibit male–female body mass dimorphism at ~1.3 [106, 107]. In modern humans, the ratio is ~1.15 [108]. Sexual dimorphism is not equal in all aspects of anatomy [5], though with regard to air sacs specifically, there is an apparent trend across the great apes. In the polygynous gorilla and orangutan (*Pongo* spp.), sexual selection has driven remarkable sexual dimorphism in vocal anatomical structures, including air sacs [2, 3]; overview in [5]. Meanwhile, chimpanzees*—*who live in multimale‐multifemale systems ‐ show almost no sexual dimorphism with regard to air sac anatomy that is not attributable to body size [37]. In behavioral domains, relationships are similarly ambiguous. In the extant African great apes, there are distinct differences in the extent and kind of sexually selected‐for competition [109, 110]. In addition, while chimpanzees and gorillas may both engage in visual and vocal displays, any contribution of air sacs to such behavior is uncertain [30].

In comparison, while there is disagreement over the extent (often aggravated by small sample sizes), there is indeed a consensus within paleoanthropology that hominins became less sexually dimorphic throughout hominin evolution, with australopith and early *Homo* males being substantially larger than females. These species likely exhibited male‐female size ratios exceeding those of extant chimpanzees [108, 111, 112, 113, 114]; (but see also [115, 116, 117]). However, an account highlighting sexual selection and competition is ultimately contingent on the delivery of future meaningful archaeological data. With regard to body dimorphism in Australopiths particularly, there is cause for hesitation. Notably, the canine teeth of australopiths—a typical marker of sexual dimorphism in primates [118, 119] – do not follow the trend otherwise indicated by species’ body size. Rather, canine dimorphism is heavily reduced in australopiths [105]. This “unusual combination” [120, p. 98] of sexually dimorphic features (low canine dimorphism, high body dimorphism) led [118, p. 219] to conclude that “the pattern of sexual dimorphism in *A. afarensis* is different from any that are observed in living humans or apes”.

As such, with regard to drawing conclusions about air sac morphology, both the young age and sex of the presumed female 3‐year‐old juvenile Dikika *A. afarensis* [7]*—*the only fossil hyoid specimen representing the time period between the disappearance of the chimpanzee‐human last common ancestor, and the Sima de los Huesos hominins at 530 kya*—*are causes of possible concern. Above, we noted considerable changes to hyoid morphology throughout the course of development. Crucially, there is no available referent for a hyoid bone in a male australopith, nor for any adult specimen. Nonetheless, several researchers have based extensive arguments on the assumption that the single australopith hyoid provides reliable clues to the morphology and evolutionary history of the genus [7, 24, 25]. The current state of paleoanthropological knowledge indicates that sexual dimorphism in australopiths cannot be straightforwardly extrapolated from extant great apes. Thus, in light of current archaeological evidence, highlighting sexual selection as explaining potential air sac loss in australopiths appears presumptuous.

## 6 Conclusions

It has long been assumed that “the bulla‐shaped [hyoid] body is the primitive condition for African apes and humans” [7, p. 300]. Conservatively, hyoid bone morphology appears preserved (i.e., “ape‐like”) in the only available australopith specimen—and derived in Middle and Upper Pleistocene hominins found in the Sima de los Huesos site, Spain, and Kebara Cave, Israel [9], and modern humans. As such, we may also—tentatively, though plausibly—extrapolate the presence or absence of air sacs. Because only five hominin hyoids—including a single Pliocene juvenile (DIK‐1/1) and a cluster of Middle to Late Pleistocene hominins—are known, any inference about earlier or in‐between (or even other contemporaneous) hominins are by necessity highly speculative at this time. In this perspective piece, we posit that the available fossil data can only provide limited support for hypotheses suggesting a verifiable link between the loss of air sacs, mating systems, and processes of sexual selection and intra‐individual competition in early hominins.

While all these factors may each have been meaningful components of the evolutionary trajectory by which air sacs were lost to ancestral hominins, the complex interplay of possible interacting factors in our evolutionary history has yet to be appreciated. We have argued that the exact functional and evolutionary significance of this anatomical trait in hominin evolution remains unclear (i.e., current evidence does not allow us to effectively discriminate between competing hypotheses concerning the relationship between hyoid morphology, air sacs, and behavior; see [28]). Future research is necessary to shine a light on this issue. In particular, the discovery and analysis of additional Pliocene and Lower Pleistocene hyoid bones, complemented by comparative developmental data from extant lesser and great apes and additional biomechanical functional modeling, may dramatically increase our understanding of relevant morphological variation in early hominins, and its possible behavioral correlates and consequences.

## Conflicts of Interest

The authors declare no conflicts of interest.

## Data Availability

Data sharing not applicable to this article as no datasets were generated or analysed during the current study.
